# Deferasirox in Patients with Chronic Kidney Disease: Assessing the Potential Benefits and Challenges

**DOI:** 10.2147/JBM.S415604

**Published:** 2023-11-28

**Authors:** Abdulqadir J Nashwan, Mohamed A Yassin

**Affiliations:** 1 Nursing Department, Hamad Medical Corporation, Doha, Qatar; 2 Department of Medical Oncology/Hematology, National Center for Cancer Care and Research, Hamad Medical Corporation, Doha, Qatar

**Keywords:** CHRONIC kidney disease, Deferasirox, chelating therapy, iron overload, hemodialysis, peritoneal dialysis

## Abstract

Chronic kidney disease (CKD) is a major global health concern, affecting millions of people worldwide. The progressive decline in kidney function often necessitates renal replacement therapy, such as hemodialysis (HD) or peritoneal dialysis (PD), to maintain a patient’s health. Iron overload, which is common in CKD patients on dialysis, can lead to severe complications, including cardiovascular disease and infections where most of the existing iron chelators are deemed unsuitable due to their suboptimal clearance in patients with compromised renal function, it becomes a significant challenge to effectively manage iron overload. Deferasirox (DFX), an oral iron chelator, has emerged as a promising treatment option for managing iron overload in these patients. However, the use of DFX comes with its unique set of challenges, such as its cost, potential side effects, and the need for close monitoring of patients, as well as the noticeable scarcity of comprehensive and rigorous clinical studies confirming its efficacy and safety of DFX. In this review, we delve into both the promising prospects and the emerging challenges associated with DFX use in managing CKD patients on HD or PD, striving for a comprehensive understanding that informs better clinical practice and patient care.

## Introduction & Background

Chronic kidney disease (CKD) is a major public health concern which affects around 10% of the global population and is the 6th fastest growing cause of death worldwide.^1^ In the United States, approximately 37 million adults, or 15% of the population, have CKD.^1^

In the United States, around 88% of patients with end-stage renal disease (ESRD) are treated with hemodialysis (HD), while 12% are treated with peritoneal dialysis (PD).^2^ Globally, HD is the most used renal replacement therapy, with approximately 3.4 million patients receiving treatment, while PD is used by approximately 11% of the ESRD population.^2^

Iron overload is a common complication in patients with CKD, particularly those on HD or PD.^3^ It is associated with increased risk of cardiovascular disease, infections, and other complications. A recent systematic review and meta-analysis^4^ estimated the pooled prevalence of severe and mild to moderate hepatic iron overload quantified by MRI was 0.23 and 0.52, respectively. The review revealed a high prevalence of severe hepatic iron overload in patients with ESRD treated by HD.^4^

There has been some controversy over the use of DFX in CKD patients due to concerns over its safety and efficacy. While DFX has been shown to effectively reduce iron overload in CKD patients, its use in this population is still not fully understood. Some studies have suggested that DFX may increase the risk of renal impairment, while others have found no significant adverse effects on kidney function. Additionally, there have been concerns over the potential interactions between DFX and other medications commonly used in CKD patients. There is also some controversy over the optimal dosing of DFX in CKD patients, with some studies suggesting that lower doses may be safer and more effective than higher doses. Therefore, this review highlights the potential benefits and challenges associated with the use of DFX in patients with CKD on HD or PD.

## Review

### Potential Benefits

#### Efficacy in Reducing Iron Overload

Deferasirox (DFX) has demonstrated efficacy in reducing iron overload or hemosiderosis in various clinical settings, including in patients with transfusion-dependent thalassemia and sickle cell disease.^5^ Its mechanism of action involves binding to excess iron and promoting its excretion in the feces, thereby reducing the risk of iron-related complications.^6^ This potential to mitigate iron overload makes DFX an attractive treatment option for CKD patients on dialysis, who often receive frequent blood transfusions and intravenous iron therapy to manage anemia.^6^

### Oral Administration

One of the key advantages of DFX is its oral formulation, which offers patients a more convenient alternative to parenteral iron chelators, such as deferoxamine.^7^ Oral administration may improve patient adherence and enhance their overall quality of life.^7^ Moreover, the once-daily dosing regimen of DFX is an added benefit, further simplifying the treatment process for patients on dialysis.^7^

### Preservation of Kidney Function

Emerging evidence suggests that DFX may have nephroprotective effects in animal models.^8^ Iron chelation therapy with DFX has been associated with improved kidney function in patients with transfusion-dependent anemias.^9^ Although the exact mechanisms are not fully understood, DFX’s ability to reduce iron deposition in the kidneys may contribute to preserving renal function.^9^ Consequently, DFX may offer additional benefits for CKD patients on dialysis by slowing the progression of renal decline.

## Challenges and Concerns

### Limited Data in CKD Patients on Dialysis

Despite the potential benefits of DFX, there is a limited amount of data regarding its use in patients with CKD on HD or PD. Most studies have focused on patients with transfusion-dependent anemias, and the evidence for DFX in CKD patients remains scarce. Therefore, more research is needed to confirm the safety and efficacy of DFX in this population, as well as to establish appropriate dosing guidelines.

Maker et al^10^ evaluated the safety and pharmacokinetics of DFX, an iron chelator, in eight hemodialysis-dependent patients with chronic kidney disease (CKD) who were receiving intravenous iron therapy. Two doses of DFX were administered (10 mg/kg and 15 mg/kg) either acutely or at steady state.^10^ The results showed that a dose of 10 mg/kg was insufficient to achieve therapeutic plasma concentrations, while 15 mg/kg-maintained plasma concentrations well above the therapeutic range.^10^ However, the observed plasma concentration was higher than expected for this dose, highlighting the importance of profiling drugs in specific patient groups such as CKD with iron overload.^10^ No adverse clinical events were observed in the study.^10^

On the other hand, Chen et al^11^ evaluated the efficacy, safety, and tolerability of DFX, an iron chelating agent, in eight HD patients with iron overload caused by transfusion-induced renal anemia. DFX treatment (15 mg/kg/day) caused significant reductions in transferrin saturation (TSAT) and ferritin levels and increases in unsaturated and total iron-binding capacity.^11^ Although there were no significant changes in hematocrit or erythropoietin levels, monthly transfusion volumes decreased significantly.^11^ DFX was generally well-tolerated, with common adverse effects being nausea, vomiting, diarrhea, and abdominal pain.^11^ The study concludes that DFX is effective, safe, and tolerable in improving iron metabolism in HD patients with iron overload.

Moreover, Yii et al^12^ reported a case of 54-year-old male with b-thalassemia major and end-stage renal disease (ESRD) was managed with continuous ambulatory peritoneal dialysis (CAPD) and required management of iron overload despite not being able to receive transfusions. DFX was administered orally at a dose of 1000 mg/day, and a total of 12.7L of peritoneal dialysate was collected over a 24-hour period with low levels of iron seen in the fluid.^12^ Over a 6-month period, serum ferritin levels decreased from 3869μg/l to 1545μg/l, and there were no episodes of peritonitis.^12^ These findings suggest that DFX may be a safe and effective agent for iron removal in iron-overloaded CAPD patients with relatively low dialysate iron levels, which reduces the risk of iron-dependent infections.

Another case reported a patient with ESRD secondary to sickle cell nephropathy who developed recurrent symptomatic hypocalcemia while receiving DFX therapy for iron overload from long-term blood transfusions.^13^ As per the authors, this is the first reported case of this complication with DFX therapy in a patient with ESRD, highlighting the need for caution and close monitoring of electrolyte levels in such patients.^13^

Furthermore, patients with transfusion-dependent thalassemia and ESRD are at risk of iron overload, which can be managed by iron chelating agents such as DFX. A report by Beydoun et al^14^ described two thalassemia patients on hemodialysis who were treated with DFX, resulting in a decrease in ferritin levels without any significant adverse events except for asymptomatic hypocalcemia in one patient. The dose used (up to 25 mg/kg/day) was higher than previously reported, indicating that DFX may be safe and effective for iron overload management in thalassemia patients with ESRD.^14^ Further studies are needed to confirm these findings.

A recent study by Bekhechi et al^15^ evaluated the efficacy of DFX on reducing liver iron concentration (LIC) in 31 dialysis patients (30 HD, and 1 PD) with secondary hemosiderosis quantified by magnetic resonance imaging (MRI). The chelation therapy resulted in a significant reduction in LIC, mean ferritin level, and a gain in mean hemoglobin and albumin levels.^15^ The therapeutic response was influenced by the cause and degree of overload.^15^ These findings suggest that DFX, prescribed at a dose of 10 mg/kg/day, can be effective in reducing iron overload in dialysis patients and may have additional benefits in improving anemia and hypoalbuminemia.^15^

Table 1 presents a comprehensive overview of significant research findings pertaining to the utilization of DFX in managing patients with CKD who are undergoing HD or PD.

**Table 1:** Summary of Key Findings on the Use of DFX in Patients with Chronic Kidney Disease on Hemodialysis or Peritoneal Dialysis

Authors	Year of Publication	Country	Study Design	Number of Patients (Age)	HD vs PD	DFX Dosage/Frequency	Main Findings	Adverse Events
Maker et al^10^	2013	Australia	Pilot study (prospective)	8	HD	Two doses tested: 10 mg/kg and 15 mg/kg. Administered either acute (once daily for 2 days) or steady state (once daily for 2 weeks)	A dose of 10 mg/kg did not achieve a therapeutic plasma concentration, whereas a dose of 15 mg/kg maintained plasma concentration well above the therapeutic range.	No adverse clinical events were observed.
Chen et al^11^	2015	Taiwan	Retrospective	8	HD	15 mg/kg/day	DFX administration led to significant reductions in TSAT and ferritin and significant increases in UIBC and TIBC. No significant differences were noted in hematocrit values or EPO requirements post-treatment, but significant reductions were observed in average monthly transfusion volumes.	Nausea, vomiting, diarrhea, and abdominal pain.
Yii et al^12^	2018	Australia	Case report	1 (Age 54; Male)	PD (patient with b-Thalassemia major developed ESRD)	1000 mg/day	Over a 6-month period, the patient’s serum ferritin fell from 3869μg/l to 1545μg/l. Iron levels in the peritoneal dialysate were lower than expected, suggesting that DFX may be a safe agent for iron removal in iron-overloaded PD patients.	No episodes of peritonitis were reported.
Yusuf et al^13^	2008	USA	Case report	1 (Age 43; Female)	PD (ESRD secondary to sickle cell nephropathy)	1500 mg/day (corresponding to 20 mg/kg for her weight)	The patient responded well to DFX therapy, with a progressive decrease in serum ferritin levels over several months.	Recurrent symptomatic hypocalcemia leading to periorbital paresthesias and mental slowing.
Beydoun et al^14^	2016	Lebanon	Letter to the editor with 2 cases	2 (A: 50 y; Male); (B: 59 y; Female)	HD (A: β-thalassemia major); (B: transfusion-dependent β-thalassemia intermedia)	Initially 15 mg/kg/day, gradually increased to 25 mg/kg/day	For patient A, ferritin levels significantly decreased from 3846 to 1447 ng/mL over 11 months. For patient B, ferritin levels showed only a mild improvement initially before increasing again.	Patient A had an episode of asymptomatic hypocalcemia
Bekhechi et al^15^	2023	Algeria	Longitudinal Study	31	30 HD 1 PD	10 mg/kg/day	Chelation with DFX resulted in a significant reduction in liver iron burden and ferritin level, as well as an increase in mean hemoglobin and albumin levels. The therapeutic response was influenced by the cause and degree of iron overload.	Nausea, vomiting, diarrhea, and abdominal pain.

**Abbreviations**: UIBC, Unsaturated Iron-Binding Capacity; TSAT, Transferrin saturation; TIBC, Total Iron Binding Capacity; EPO, Erythropoietin.

### Adverse Effects

The use of DFX is not without potential risks. Common side effects include gastrointestinal symptoms, such as abdominal pain, nausea, and diarrhea. More serious adverse effects, although rare, can include hypocalcemia,^13^,^14^ renal impairment, hepatic dysfunction, and hematologic abnormalities.^16^ These potential complications may be of particular concern in CKD patients on dialysis, who often have multiple comorbidities and are more susceptible to drug-related adverse events.^16^ Careful patient monitoring and dose adjustments may be necessary to minimize the risks associated with DFX therapy.

### Drug Interactions

DFX is metabolized by the liver and may interact with other medications commonly prescribed to CKD patients, such as phosphate binders, antihypertensive drugs, and erythropoiesis-stimulating agents.^17^ These interactions can potentially alter the effectiveness of the medications and increase the risk of adverse effects.^17^ Therefore, healthcare providers must carefully consider potential drug interactions and adjust treatment regimens accordingly to optimize patient outcomes.

### Cost and Access

The cost of DFX can be a significant barrier to its widespread adoption in CKD patients on dialysis.^18^ DFX is generally more expensive than traditional parenteral iron chelators, which may limit its accessibility, particularly for patients in low-resource settings or those without adequate insurance coverage. Efforts to address cost-related concerns and improve access to DFX will be crucial for ensuring that more patients can benefit from this therapy.

## Conclusion

DFX represents a promising treatment option for managing iron overload in patients with CKD on hemodialysis or peritoneal dialysis. Its oral administration, potential nephroprotective effects, and demonstrated efficacy in reducing iron overload make it an attractive alternative to traditional parenteral iron chelators. However, several challenges, including limited data in CKD patients on dialysis, potential adverse effects, drug interactions, and cost concerns, must be addressed before DFX can be widely adopted in this population. Future research should focus on evaluating the safety and efficacy of DFX in CKD patients on dialysis, as well as on exploring strategies to mitigate potential risks and improve patient outcomes. Additionally, efforts should be made to increase access to DFX and to develop patient-centered treatment strategies that promote adherence and enhance the overall quality of life for individuals with CKD on dialysis. By addressing these challenges, DFX may ultimately become an integral component of the comprehensive management of iron overload in patients with CKD on HD or PD.

## Data Sharing Statement

All data generated or analyzed during this study are included in this article. Further enquiries can be directed to the corresponding author.
